# MiR‐33‐5p‐opioidergic Signaling Regulates Cognitive Impairment Induced by Bile Duct Ligation in Rats: Ameliorative Role of Naloxone

**DOI:** 10.1002/brb3.71105

**Published:** 2025-11-25

**Authors:** Mohadeseh Rahimi‐vala, Behrang Alani, Ali Arjmand, Tahere Mazoochi, Abolfazl Ardjmand

**Affiliations:** ^1^ Physiology Research Center, Institute for Basic Sciences Kashan University of Medical Sciences Kashan Iran; ^2^ Autoimmune Diseases Research Center Kashan University of Medical Sciences Kashan Iran; ^3^ Department of Applied Cell Sciences, School of Medicine Kashan University of Medical Sciences Kashan Iran; ^4^ Department of Biology, Faculty of Basic Sciences University of Guilan Rasht Iran; ^5^ Gametogenesis Research Center Kashan University of Medical Sciences Kashan Iran; ^6^ Department of Physiology, School of Medicine Kashan University of Medical Sciences Kashan Iran

**Keywords:** bile duct ligation, cholestasis, cognition, miR‐33‐5p, opioids, rat

## Abstract

**Objective:**

The present study investigated the involvement of miR‐33‐5p‐opioidergic signaling in the regulation of cognitive impairment induced by bile duct ligation (BDL) in rats, with an emphasis on the ameliorative role of naloxone.

**Methods:**

Among the four groups of Wistar rats (control, sham, BDL, and BDL + Naloxone [Nalx]), the common bile duct was occluded only in the BDL groups. Fourteen days after BDL induction and following completion of the analysis of the liver function tests of alkaline phosphatase, alanine aminotransferase, aspartate transaminase, direct bilirubin, total bilirubin, gamma‐glutamyl transpeptidase (GGT), and lactate dehydrogenase, the cognitive tests and electrophysiological field potential responses were recorded. Then, the hippocampus was assessed for the expression level of miR‐33‐5p using PCR. Ultimately, a histopathological study of the liver and hippocampus was carried out.

**Results:**

The findings revealed that except for GGT (*p* = 0.1935), all liver function tests were elevated (*P* = 0.0001) following BDL. The impaired inhibitory avoidance memory (*P <* 0.0001) and plasticity (*P* < 0.0001) and the hippocampal overexpression of miR‐33‐5p following BDL (*P* <0.0001) were ameliorated by Nalx. In a qualitative histopathological study of the liver and hippocampus, Nalx attenuated the BDL‐induced changes.

**Conclusions:**

It is concluded that the BDL‐mediated dysfunction has a molecular, electrophysiological, and histopathological basis and might be regulated via miR‐33‐5p and opioidergic signaling. The study emphasizes the potential ameliorative role of Nalx on biliary and nervous system pathologies.

## Graphical abstract

Bile‐duct ligation (BDL)‐induced memory impairment has a molecular, electrophysiological and histopathological basis and might be regulated by miR‐33‐opioid signaling. The modulation of miR in the brain may be a novel therapeutic approach for relieving neuropathological problems in cholestatic cases, and individuals with cholestasis may benefit from new therapeutic approaches that focus on this brain circuit in terms of cognitive and synaptic plasticity.

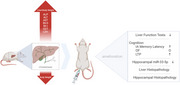

## 1 Introduction

Hepathobiliay pathologies are identified to adversely affect the functions of remote systems, including the nervous system (D'Mello and Swain 2011). Several studies have shown that hepatic pathologies may lead to cognitive impairments and, in advanced stages, to hepatic encephalopathy (HE) (Butterworth 2013; Claeys et al. 2021; Ohikere and Wong 2024; Prasad et al. 2007). HE is a complex neurological disorder that results from severe liver illness (Sepehrinezhad et al. 2023). Despite the controversy on the pathogenesis of HE (Ren et al. 2025), it is characterized by hyperammonemia, aberrant electroencephalographic signals, and varying degrees of disruption in sensorimotor and cognitive functioning (Lu 2023). Hyperammonemia brought on by HE changes the brain's astrocytic glutamate metabolism, which contributes to cognitive disorders (Jo et al. 2023). Bile duct ligation (BDL) is known as an established animal model for the investigation of HE that can similarly impair cognition (Sheen et al. 2016).

A thorough analysis of the literature using various learning and memory models demonstrates that memory is impaired in HE by various mechanisms involving different brain areas. For instance, in animals, novel object recognition (Cho et al. 2020; Ganjalikhan‐hakemi et al. 2023; Leke et al. 2013), spatial recognition memory (Javadi‐Paydar et al. 2013), and inhibitory avoidance memory (Bayat et al. 2022; Mohammadian et al. 2019; Nasehi et al. 2013; Zarrindast et al. 2020) and similarly in humans (Visitchanakun and Leelahavanichkul 2018). Moreover, long‐term potentiation (LTP), a neurophysiological event that is regarded as the hallmark of memory and learning, occurs in the adult brain and is largely responsible for neurogenesis and neuronal plasticity in the hippocampus (Anand and Dhikav 2012). Reportedly, LTP is altered in HE animals (França et al. 2019; Mohammadian et al. 2019; Sun et al. 2019).

In addition, it is presumed that the opioidergic system, via the μ‐opioid receptor (Hosseini‐Sharifabad et al. 2016; Nava‐Mesa et al. 2013), plays a role in the pathogenesis of cholestasis (Bergasa 2018) and also modulates memory processes in cholestatic animals (Afshari et al. 2018; Nasehi et al. 2013; Zarrindast et al. 2012). A previous study reported that endogenous opioids increase after BDL (Ahmadi et al. 2015). Moreover, some reports demonstrating the physiopathological relationship between the opioidergic system and HE are further augmented by the evidence that Naloxone (Nalx) alleviates the symptoms of human HE (Eguchi 2004; Jiang et al. 2010;) especially uremic and cholestatic pruritus (Anand 2013; Legroux‐Crespel et al. 2004; Peer et al. 1996; Terg et al. 2002; Wolfhagen et al. 1997), and symptoms of animal fulminant hepatic failure (Yurdaydin et al. 1995). In this regard, the effectiveness of Nalx to alleviate pruritus following bile duct obstruction is also reported, in which the hypersensitivity of mu‐opioid receptors on C‐fibers to itching stimuli is caused due to higher concentrations of biliary acidic metabolites (Włodyka et al., 2025). Thence, the aforementioned findings that Nalx reverses the BDL‐induced amnesia in animals (Nasehi et al. 2013) and that Nalx facilitates the consciousness recovery in cases with HE (Ghiassy et al. 2017; Jiang et al. 2010) collectively signify the bodily and cognitive restorative functions of the Nalx (Nasehi et al. 2016).

HE is a stage of mental state associated with brain injury or dysfunction. Similarly, in reaction to injury or damage to the brain, proteins that protect the brain from insult are produced. MicroRNAs (miRs) regulate the synthesis of the mentioned proteins, at least partially (Visitchanakun and Leelahavanichkul 2018). Numerous studies show that miRs are short non‐coding RNAs that regulate gene expression and have received considerable verification as potential biomarker candidates in a variety of disorders (Garcia‐Martínez et al. 2016; Korte and Schmitz 2016; Orzeł‐Gajowik et al. 2021; Wei et al. 2017). Although plenty of evidence supports the function of miRs in controlling opioid signaling (Gao et al. 2010; He et al. 2010; Vastegani et al. 2022; Wu et al. 2009; Zheng and Chu et al. 2010), suggests that miRs are important modulators of opioid‐related cognitive abilities (Guo et al. 2002; He et al. 2010; Vastegani et al. 2022), and the function of miRs has been extensively studied in learning and memory processes, little is known about their specific regulatory function in the cognitive impairment induced by HE through miR‐33.

*MiR‐33*, which is abundantly expressed in the brain and has a crucial role in lipid metabolism and cholesterol homeostasis, is essential for neuronal activities (Kim et al. 2015; Najafi‐Shoushtari et al. 2010; Rayner et al. 2010). *MiR‐33* under‐expression in the brain of rodents results in the expression of *ATP*‐binding cassette transporter *A1* (*ABCA1*), ApoE lipidation, and a decrease in intrinsic brain amyloid beta (Aβ) level. It is hypothesized that abnormalities in the metabolism of Aβ peptides initiate destructive processes that can lead to Alzheimer's disease (Haass and Selkoe 2007). Furthermore, in an animal model of Alzheimer's disease, the pharmacologic blockade of cerebral miR‐33 significantly reduced cortical Aβ (Mohammed et al. 2017), suggesting a potential treatment role for miR‐33 in both Alzheimer's disease and a wide range of neurological/cognitive disorders (Jaouen and Gascon 2016; Kim et al. 2015; Mohammed et al. 2017; Reddy et al. 2017).

Considering the aforementioned and also our earlier research demonstrating that the hippocampal expression levels of miR‐33‐5p act as a mechanism for the opioid‐induced impairment of memory and the involvement of the μ‐opioid receptor in its modulation (Vastegani et al. 2022), the present study investigates the involvement of cognitive impairment induced by BDL via *miR‐33‐5p* opioid signaling in rats.

## 2 Methods

### 2.1 Subjects

A total of 42 Wistar rats, weighing 200–250 g, were allocated to four groups (control, sham, bile duct ligation [BDL], and BDL + Nalx). Among the four groups, the common bile duct was occluded only in the BDL groups. The rats were housed in a colony room with a 12‐h light/dark cycle and a constant temperature of 22 ± 2°C. All food and drink were available to the animals without restriction, with the exception of during the experiment times. Every attempt was made to reduce the number of animals employed in the research as well as their suffering. The 2011 Guide for the Care and Use of Laboratory Animals developed by the National Academy of Sciences' Institute for Laboratory Animal Research served as the basis for all the procedures. The study protocol (IR.KAUMS.AEC.1401.008) was approved by Kashan University of Medical Sciences (KAUMS) Research Ethics Committee (REC). For the 10% possibility of mortality (Cho et al. 2020), two more rats were assigned to the BDL and BDL+ naloxone (Nalx) groups.

### 2.2 Drugs

The rats were randomly allocated to each of the experimental groups. Nalx (Sigma, England) was dissolved in physiological saline. In the group that was administered Nalx, 2 mg/ml/kg/*i.p*. of the medicine was injected 30 min prior to the experiments. The dose of Nalx was determined from pilot studies and previously established protocols (Mariani et al. 2011; Zarrindast et al. 2020). Meanwhile, the control group received saline (1 mL/kg). Cell Signaling Technology provided anti‐rabbit HRP‐conjugated secondary antibody and primary antibodies specific to phosphor‐CREB (New York, USA).

### 2.3 Study Design

**FIGURE 1:**
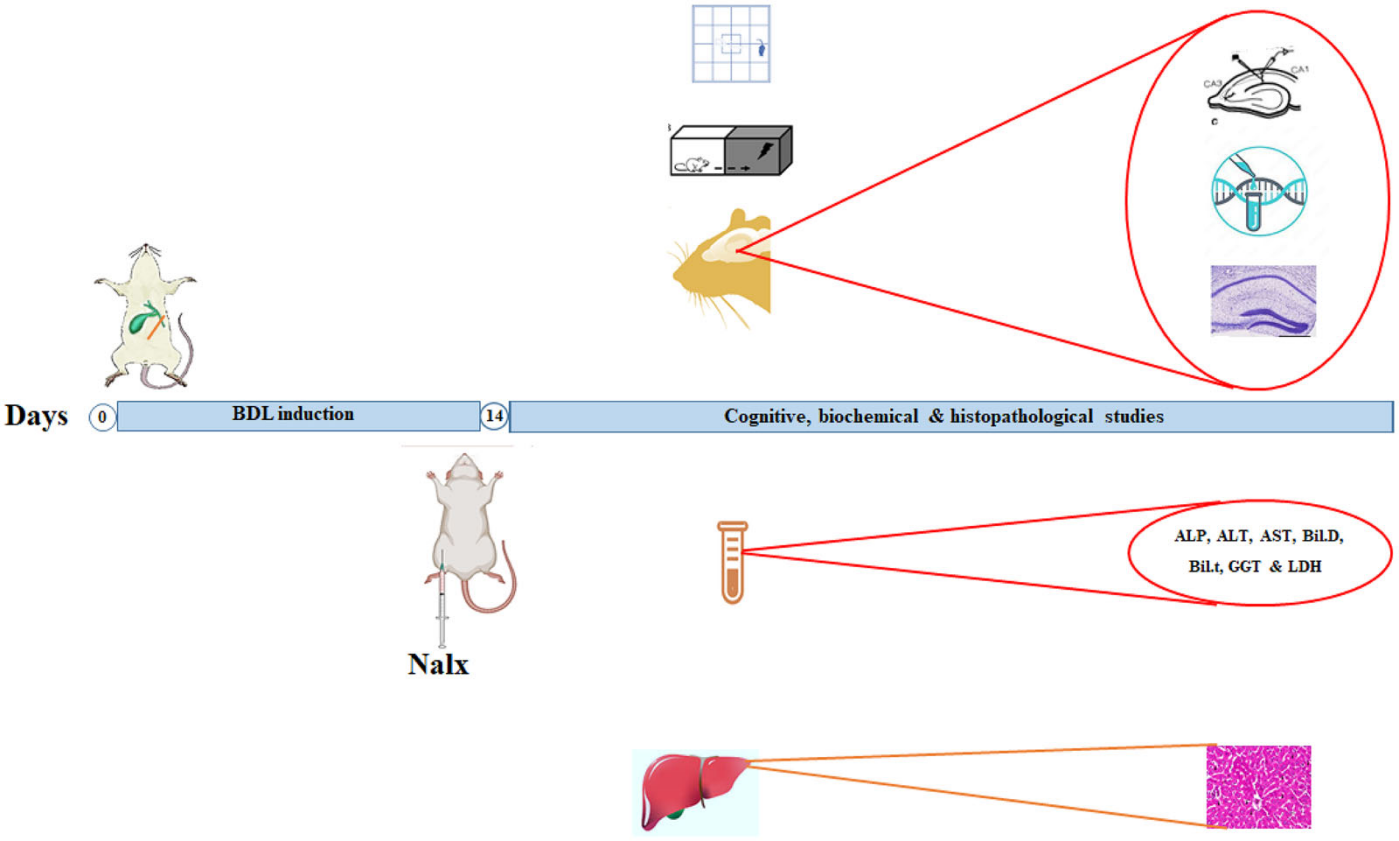
Experimental design for the study.

Fourteen days after the BDL surgery, and following the completion of the cognitive tests (inhibitory avoidance [IA] memory test, open field [OF]. and electrophysiological field potential recording), blood samples were taken for the biochemical analysis of the liver function tests. Then, after decapitation, the hippocampi were quickly removed and frozen in liquid nitrogen and kept at −80°C, as previously described (Leke et al. 2013). Simultaneously, hippocampi were extracted from an intact group without any intervention. Following this step, three distinct randomly selected hippocampi per experimental group and method were used to assess the expression levels of miR transcripts using quantitative real‐time polymerase chain reaction (PCR). Then, the distinct randomly selected liver and hippocampal samples from each experimental group were studied for qualitative histopathological assessment (Jabbari et al. 2023) (Figure 1).

### 2.4 Induction of BDL

The BDL and sham procedures were performed under sterile conditions, as previously described (Nasehi et al. 2023). In agreement with previous studies (Cho et al. 2020; Claeys et al. 2023), the HE in our study was developed 14 days after BDL, supporting their suggestion that this time point is appropriate for investigating liver disease‐related extrahepatic neurocognitive dysfunction. Two weeks after the surgery, the behavioral and electrophysiological experiments were performed in an orderly fashion for 2 days (Jo et al. 2023). In short, under ketamine (50 mg/kg)/xylazine (5 mg/kg) anesthesia, a midline abdominal incision was made, and the common bile duct was isolated from the adjacent portal vein. Next, the common bile duct was ligated with a double ligature of nylon suture (SUTURES Co. 5/0 USP, Iran), and a cut was made in between the ligatures to prevent recanalization. The rats received buprenorphine 0.1 mg/kg intraperitoneally (*i.p*.) for 72 h to prevent postoperative pain and distress and were sacrificed 14 days post‐surgery.

### 2.5 Liver Function Tests

Using blood serum samples, the biochemical analysis of the liver function tests, including alkaline phosphatase (ALP), alanine aminotransferase (ALT), aspartate transaminase (AST), direct bilirubin (Bil.D), total bilirubin (Bil.t), gamma‐glutamyl transpeptidase (GGT), and lactate dehydrogenase (LDH), was conducted according to the manufacturer's kit (KiaZist, Hamadan, Iran). The experimenter who studied the specimens was blinded to the experimental procedures of this research.

### 2.6 IA Test

The IA experiments were conducted in accordance with the procedure described in an earlier report (Jabbari et al. 2023). The IA apparatus consisted of two identical dark/light chambers (20 × 20 × 30 cm^3^), divided by a wall, with an automatically‐controlled guillotine door in the center (Technique Azma, Tabriz, Iran). The floor of the apparatus was made up of parallel stainless steel grids spaced one centimeter apart, each measuring 3 millimeters in diameter. Using an isolated stimulator, intermittent electric shocks with a frequency of 50 Hz, duration of 3 s, and intensity of 1 mA were administered to the dark chamber's grid floor. The experiment was divided into two sessions, each performed on a separate day: a training session and a memory retention (test) session that followed. Each rat was carefully placed in a white chamber during the training session, and the rat's latency to step through the guillotine door and enter the dark chamber with all four paws was measured. After the rats completely moved with all four of their paws on the grid floor to enter the dark chamber, an electric shock was delivered to the animal paw with a stimulator. With the exception of delivering an electric shock, the memory retention session was run similarly to the training session. The criterion for evaluating memory was the latency time (sec) to enter the dark chamber. A consistent experimental environment was provided for each rat, taking into account the impact of environmental factors on synaptic plasticity (Zheng and Chu et al. 2010).

### 2.7 OF Test

As previously described (Kharazmi et al. 2022), to assess the spontaneous locomotor activity, the animals were put in an OF apparatus after the training session (on the first day) only for habituation purposes, and the data were recorded after the testing session (on the second day) for 5 min in an OF chamber using a video tracking system (Technique Azma, Tabriz, Iran). The data were reported as the total horizontal distance traveled (cm).

### 2.8 In Vivo Electrophysiology

Under urethane anesthesia (1.5 g/kg, *i.p*.), the rats were fixed in a stereotaxic frame (Narishige, Japan) for electrophysiological recording, as previously described (Alinaghipour et al. 2022). Two holes (1 mm in diameter) were drilled into the skull using a dentistry drill in order to insert the stimulating and recording electrodes into the brain. Initially, a stimulating electrode was placed at the stereotaxic coordinates (3.5 mm below the dura surface, 3.4 mm lateral to the midline, and 4.2 mm posterior to the bregma) in Schaffer's collaterals, and then a recording electrode was placed at the CA1 region of the hippocampus (coordinates 2.8 mm below the dura, 2.5 mm lateral to the midline, and 3.8 mm posterior to the bregma) (Paxinos and Watson 2013). A Teflon‐coated stainless steel wire (A‐M Systems, 0.008‐inch diameter, USA), exposed only at its tip (bare tip 0.10 mm), was used to make the electrodes. Stereotaxic and electrophysiological markers were used to establish correct electrode placement. Field excitatory postsynaptic potentials (fEPSPs) were recorded from the CA1 region of the hippocampus in response to the stimulation (two sweeps/min at 30‐s intervals) of the ipsilateral to Schaffer's collateral region using special software (eProbe, ScienceBeam, Iran). When the response was stable, an input‐output curve was recorded using a variety of stimulus currents. Afterwards, the level of stimulation was adjusted to produce an fEPSPs amplitude that was 60% of the maximum response. To compare with post‐tetanus responses, baseline fEPSPs were averaged over a 30‐min period. Next, a high‐frequency stimulation (HFS) of 100 Hz was used to produce the LTP (10 bursts of 10 stimulations, stimulus length 0.2 ms, and burst intervals 10 s). At least 2 h of recordings were made following the tetanus stimulation. The changes in the pre‐ and post‐tetanus recordings' amplitude as a percentage were taken into account in the analysis of the data.

### 2.9 Determination of Rno‐miR‐33‐5p Expression Levels by Quantitative/Real‐Time PCR

For the PCR, the Hybrid‐R miRNA Kit (GeneAll, Seoul, Korea) was used to extract total hippocampus miR according to the manufacturer's recommendations. Using a Nanodrop ND 3300 spectrophotometer (Thermo Scientific, USA), the quantity and quality of the isolated miRs were assessed. After miR extraction, cDNA synthesis was performed using an RT stem‐loop miR primer kit specifically for both miR‐33‐5p and SNORD48 (endogenous control). MicroRNA expression was quantified using the Ana microRNA kit (Catalog # 1‐33‐5P) from Anacellteb (Tehran, Iran) according to the manufacturer's instructions. The reverse transcription and qPCR primer sequences are proprietary components of this commercial kit and are not publicly available due to intellectual property restrictions. The kit has been rigorously validated by the manufacturer for specificity and efficiency for each target microRNA. Quantitative polymerase chain reaction was carried out using SYBR‐Green/ROX qPCR master mix assay (Ampliqon, Denmark) by specific primers (ZistRoyesh, Tehran, Iran). All the reactions were implemented in triplicate using the LightCycler 96 Instrument (Roche Life Science), and the 2^−ΔΔCt^ method was applied for data calculation.

### 2.10 Histopathological Evaluation of the Liver and Hippocampal Samples

Finally, the animals were sedated via CO_2_ inhalation and euthanized by rapid decapitation. The liver and hippocampi were then collected for qualitative pathological examination (Jabbari et al. 2023). The specimens were preserved in 10% neutral buffered formalin, dehydrated in graded ethanol (70%–100%), then underwent two changes of xylol cleaning and ultimately embedded in melted paraffin wax. Afterwards, the 6‐µm‐thick microtome slices were made and stained with hematoxylin/eosin and Masson's trichrome method. Hematoxylin‐eosin and Masson's trichrome staining were used for the liver, and hematoxylin‐eosin for the hippocampus. Liver pathology was assessed based on biliary duct fibrosis, inflammation, necrosis, and hyperplasia, while hippocampal pathology was evaluated by cell morphology, cytoplasmic vacuolation, and nuclear pyknosis. The specimen examiner was not informed of the experimental methods used in this study.

### 2.11 Statistical Analysis

After assessing the normality of the data and equality of variances using the Brown‐Forsythe test and the Shapiro–Wilk test, respectively, a one‐way ANOVA was performed to analyze the behaviors. Post‐hoc comparisons were made using Tukey's test. The one‐way ANOVA was employed to examine the effects of the behavioral tests, gene expression, and protein levels. The statistical significance was set as *P* < 0.05. Plotting and data analysis were performed with GraphPad Prism, version 9.5.1. Data sharing upon reasonable request is the responsibility of the corresponding author.

## 3 Results

### 3.1 BDL Induction Altered the Post‐operative Body and the Ratio of Liver to Body Weights

**FIGURE 2:**
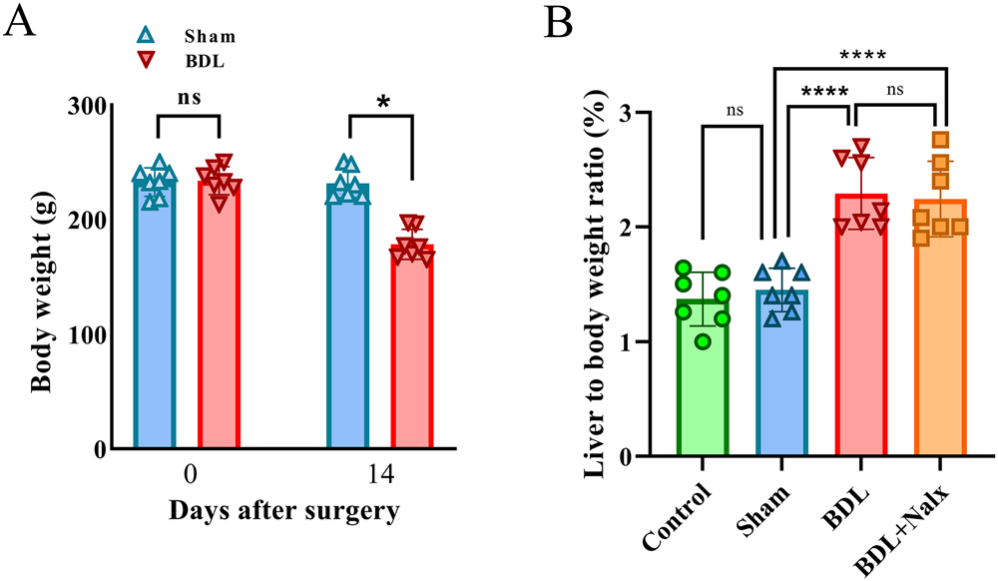
**(A)** Changes in body weight between sham and BDL groups, 14 days after surgery (*n* = 7 for each group) and **(B)** The ratio of liver to body weight (%) in experimental groups. Values are presented as mean ± SD, (*n* = 7 for each group). BDL, bile duct ligation. Nalx, naloxone. ns, a non‐significant effect. ^****^ *p* < 0.0001 and *p < 0.05 vs. sham group.

Although 14 days after BDL surgery, the preoperative body weight of the sham and BDL rats was not significant [*t* (12)= 0.1952, (*p* = 0.8485)], the postoperative body weight significantly decreased in the BDL group compared to the sham group [*t* (12)= 7.714, (*P* < 0.0001)], (Figure 2A).

Moreover, 14 days after BDL surgery, the ratio of liver to body weight (%) in the control and sham rats was not significant [*t* (3, 24) = 23.21, (*P* < 0.0001)]. Meanwhile, the ratio of liver to body weight (%) significantly increased in the BDL group compared to the sham group (*P* < 0.0001). Based on the detection of these parameters, we inferred that the induced BDL model had severe liver failure (Figures 2A and 2B).

### 3.2 BDL Induction Caused Alteration of the Liver Function Tests

**FIGURE 3:**
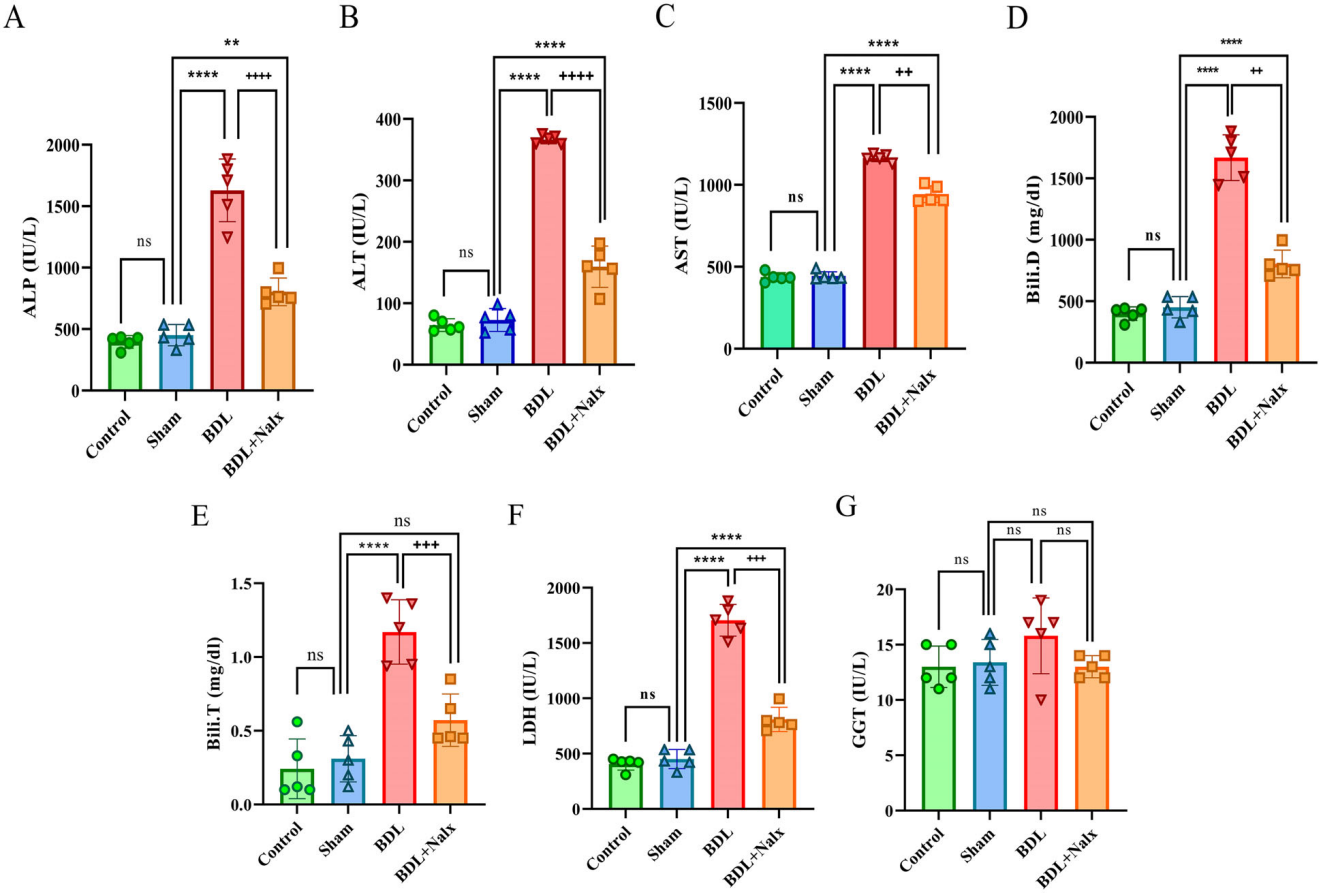
**(A–G)** The effect of BDL on liver function tests (serum ALP, ALT, AST, Bil.D, Bil.t, GGT and LDH) in BDL‐induced rats. Values are presented as mean ± SD, (*n* = 5 for each group). **Abbreviations**: ALP, alkaline phosphatase; ALT, alanine aminotransferase; AST, aspartate aminotransferase; BDL, bile duct ligation; Bil.D, direct bilirubin; Bil.t, total bilirubin; GGT, Gamma‐glutamyl transpeptidase; LDH, lactate dehydrogenase; Nalx, naloxone. ^****^ *p* < 0.0001and ^**^ *p* < 0.01 vs. sham group. ^++^p < 0.01, ^+++^p < 0.001, and ^++++^p < 0.0001 vs BDL group. ns, a non‐significant effect.

After the termination of the cognitive tests, the effect of BDL was examined on the liver function tests. The results of the one‐way ANOVA showed a significant increase in the liver function test results in the BDL group, including ALP [*F* (3, 16) = 73.86, *P* < 0.0001], ALT [*F* (3, 16) = 242.1, *P <* 0.0001], AST [*F* (3, 16) = 73.86, *P* < 0.0001)], Bil.D [*F* (3, 16) = 119.6, *P* < 0.001], Bil.t [*F* (3, 16) = 24.59, *P* = 0.0001] and LDH [*F* (3, 16) = 167.8, *P* = 0.0001], but not GGT [*F* (3, 16) = 1.769, *P* = 0.1935] compared to the sham group. Nonetheless, Tukey's post‐hoc analysis showed that the administration of Nalx in the BDL + Nalx group (2 mg/kg) significantly attenuated BDL‐induced liver damage by decreasing the activities of ALP (*P* < 0.0001), ALT (*P* < 0.0001), AST (*P* < 0.0001), direct bilirubin (*P* < 0.0001), total bilirubin (*P* < 0.001), and LDH (*P* < 0.001) compared to the BDL group. The obstruction of the biliary system damages the hepatocytes and raises the levels of serum ALT, AST, ALP, Bil.D, Bil.T (Ganjalikhan‐hakemi et al. 2023; Mohammadian et al. 2019), and GGT (Škvareninová and Kostecká 2023; Tamnanloo et al. 2023). Also, compared to the sham group, the levels of Bil.t and Bil.D were higher in the BDL group, which suggests that bilirubin was not excreted due to the ligation of the bile duct. Bilirubin, as one of the components of bile acid, is made from hemoglobin breakdown in the blood and is considered a main index to evaluate liver dysfunction (López‐Velázquez et al. 2014) (Figures 3A–G).

### 3.3 BDL Induction‐impaired IA Memory

**FIGURE 4:**
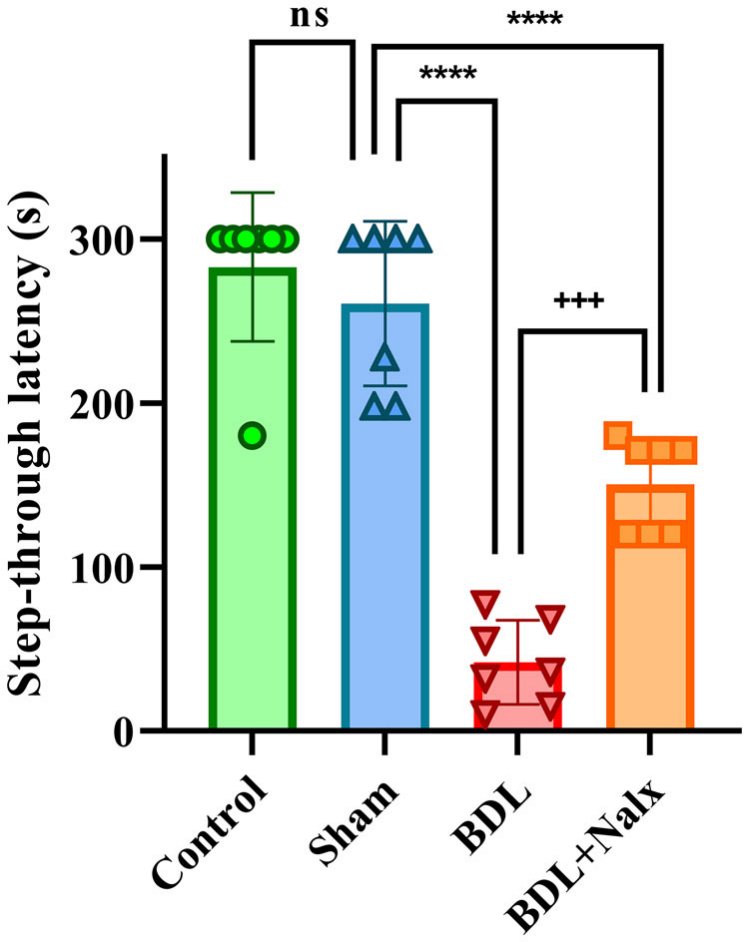
The effect of BDL on step‐through latency (sec) in an inhibitory avoidance memory task. Values are presented as mean ± SD, (*n* = 7 for each group). **Abbreviations**: BDL: bile duct ligation; Nalx, naloxone; ns, a non‐significant effect. ^****^ *p* < 0.0001, vs. sham group. ^+++^p < 0.001 vs. BDL group.

Fourteen days after the surgery, the effect of BDL on IA memory was evaluated. The results of the one‐way ANOVA showed a significant difference among the groups [*F* (3, 24) = 56.98, *P <* 0.0001]. Moreover, Tukey's post‐hoc analysis showed a significant difference among the BDL, BDL + Nalx, and sham groups (*P <* 0.0001), and also between the BDL + Nalx and the BDL groups (*P <* 0.0001) (Figure 4).

### 3.4 BDL Had no Effect on Spontaneous Locomotor Activity

**FIGURE 5:**
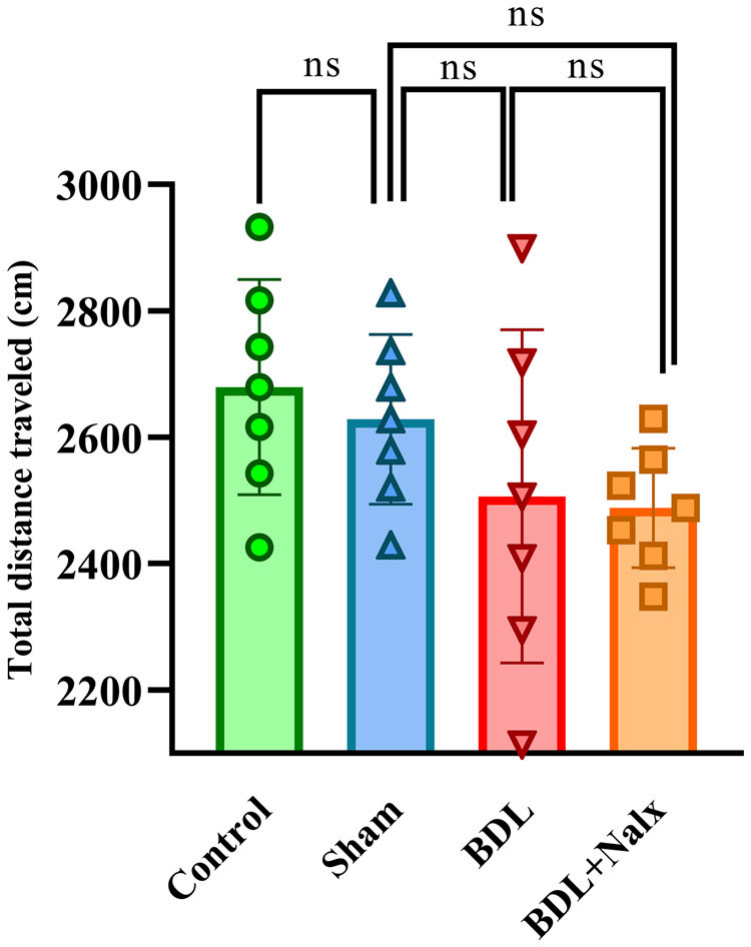
The effect of BDL on spontaneous locomotor activity in open‐field task. Values are presented as mean ± SD, (*n* = 7 for each group). **Abbreviations**: BDL, bile duct ligation; Nalx, naloxone; ns, a non‐significant effect.

Following the termination of IA memory testing (on the second day), the effect of BDL on locomotor activity of the animals was evaluated in the OF for 5 min. The results of the one‐way ANOVA showed no differences among the groups [*F* (3, 24) = 1.936, *p* = 0.1507]. (Figure 5).

### 3.5 BDL Altered the Baseline Synaptic Transmission in the Hippocampal Schaffer Collateral/CA1 Circuit

**FIGURE 6:**
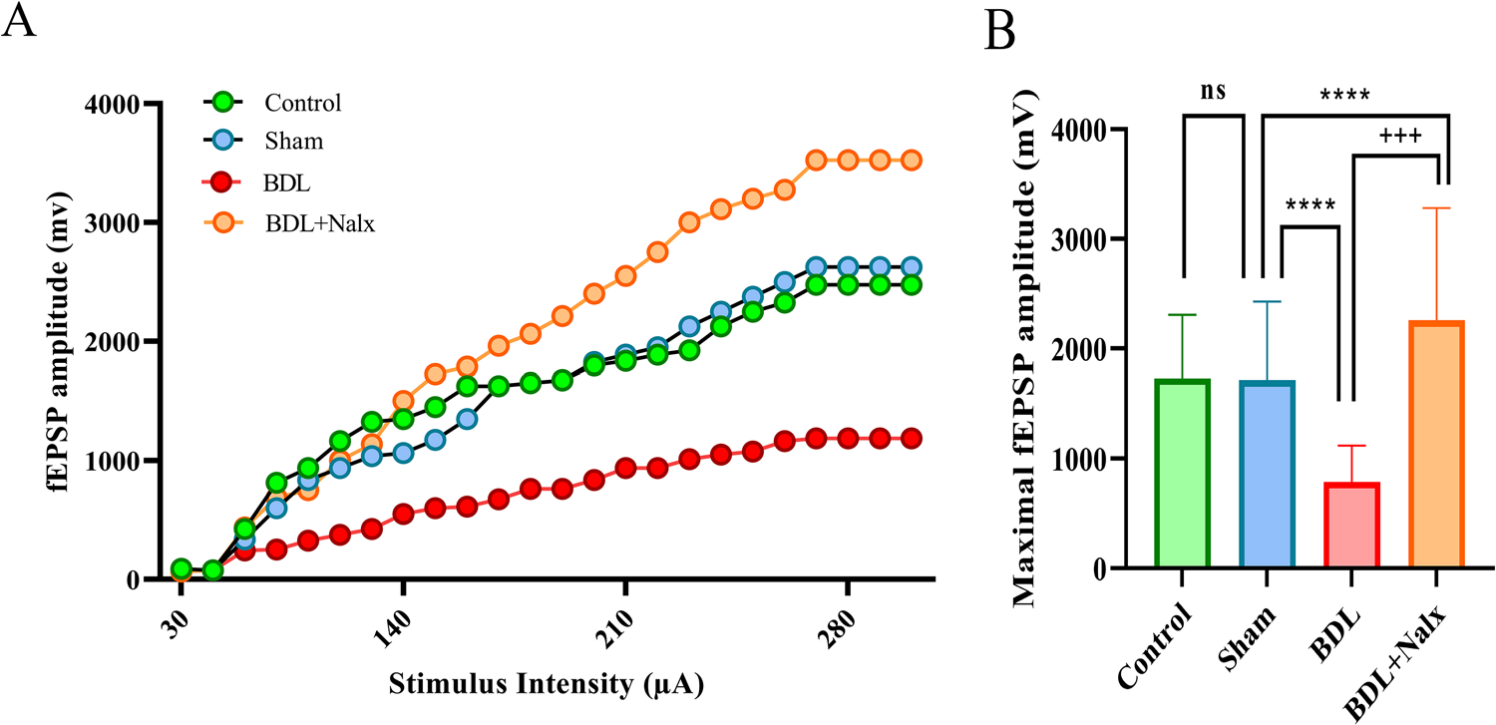
Baseline Synaptic transmission in the hippocampal Schaffer collateral/*CA1* circuit. **(A)** The input/output curve and **(B)** The comparison of maximal fEPSP amplitude (mv). Values are expressed as mean ± SD, (n = 3 for each group). ****p < 0.0001 vs. sham group and ^+++^p < 0.001 vs. BDL group. **Abbreviations**: BDL, bile duct ligation; Nalx, naloxone; ns, a non‐significant effect.

We evaluated the basal synaptic responses in the Schaffer‐collateral/CA1 circuit by progressively increasing the stimulus intensity (90–300 µA) and recording the fEPSP in the CA1 region. The input/output curves of the BDL group, but not the BDL + Nalx group, revealed a clear trend of decrease by its downward and rightward shift compared to the sham group. Also, the comparison of maximal fEPSP amplitudes revealed that the maximal stimulation strength responses were significantly lower in the BDL group (788.352 ± 328.5 mV, *P* < 0.0001), but not in the BDL + Nalx group (2257 ± 1025 mV), compared to the sham group (1731.25 ± 577.1 mV, *P* < 0.0001). (Figures 6A and 6B).

### 3.6 BDL‐induced LTP Impairment was Reversed by Nalx

**FIGURE 7:**
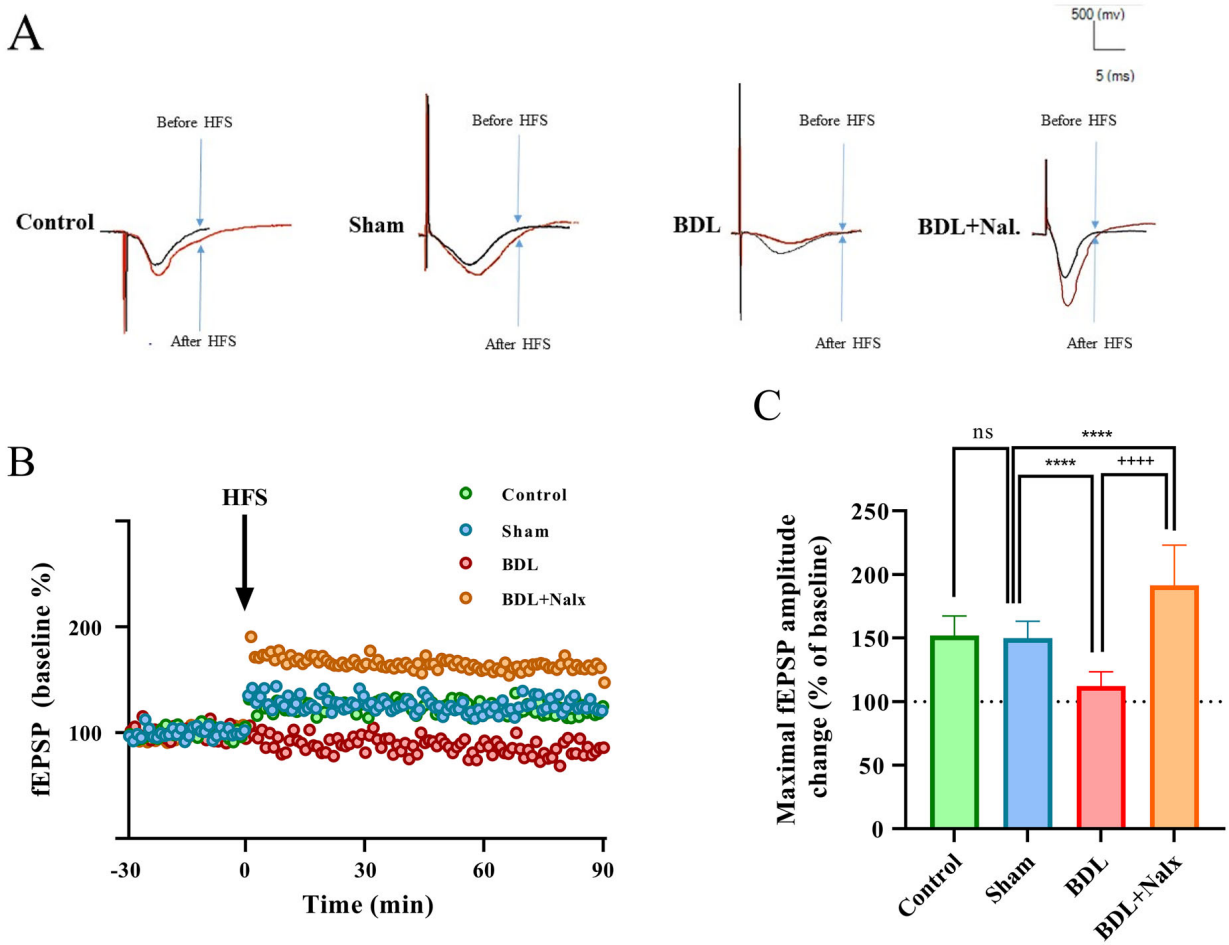
**(A)** The sample traces of responses and level of LTP induction. The beneficial effects of Nalx (2 mg/kg/*i.p*.) administration on the impairment of LTP in BDL animals, **(B)** The plots of normalized fEPSP amplitudes. The values are expressed as percent change of fEPSP slope relative to the baseline after HFS stimulation; the downward arrows indicate HFS delivery, and **(C)** The mean of 60‐min percentage change of fEPSP slope after HFS in each group. Abbreviations: BDL, bile duct ligation; HFS, high frequency stimulation; LTP, long‐term synamptic plasricity; Nalx, naloxone. Values are shown as means ± SD. ^****^ *p* < 0.0001 vs sham and ^++++^p < 0.0001 vs BDL group. ns: a non‐significant effect.

As shown in the sample traces and plots of normalized *fEPSP* amplitudes, LTP was induced by the HFS delivery to the Schaffer collateral‐CA1 circuits in the control, sham, and BDL+ Nalx groups, but not in the BDL group. In addition, the mean alterations of fEPSP (percentage from baseline) were calculated for 90 min after the delivery of HFS, and the high values obtained for the sham and BDL + Nalx groups (152.0 ± 15.28% and 191.4 ± 31.67%, respectively), comprising statistically significant differences (P < 0.0001 and P < 0.0001, respectively), further confirmed the successful induction of LTP in these groups. In contrast, the impairments of LTP induction were also verified again in the BDL group with the low and comparable value of 112.2 ± 11.23% for the mean percentage of changes in fEPSPs after HSF, which was lower than in the control, sham, and BDL + Nalx groups (*P* < 0.0001), and there was also a significant difference between the BDL and BDL + Nalx groups in this regard (*P* < 0.0001). Furthermore, the magnitude of LTP was found to have no significant difference between the control and sham groups (*P* = 0.8437) (Figures 7A, 7B, and 7C).

### 3.7 BDL Induction Overexpressed the Hippocampal miR‐33‐5p Level

**FIGURE 8:**
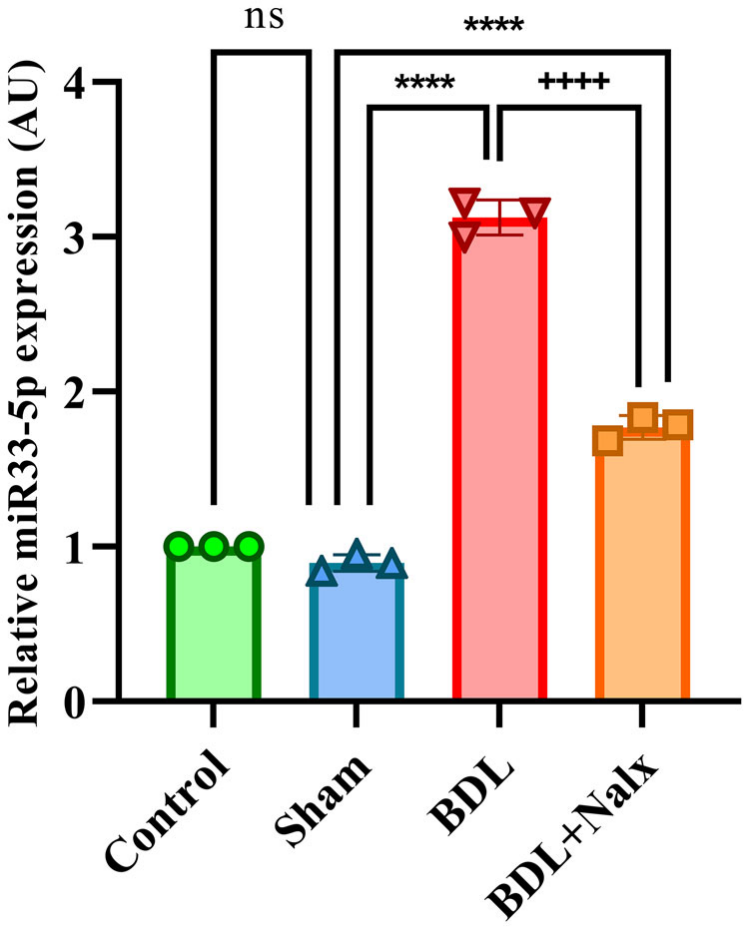
The hippocampal miR‐33‐5p expression in BDL‐induced memory impairment. Values are expressed as mean ± SD, (*n* = 3 for each group). ^****^ *p* < 0.0001 vs sham group, ^++++^p < 0.0001 vs. BDL group. **Abbreviations**: BDL, bile duct ligation; Nalx, naloxone; ns, a non‐significant effect.

Fourteen days following the termination of the experiments, we examined the expression levels of miR‐33‐5p in the hippocampus of the BDL‐induced rats. The results of the one‐way ANOVA showed the hippocampal overexpression of *miR‐33‐5p* in the BDL and BDL+ Nalx groups compared with the sham group [*F*(3, 8) = 577.8, (*P* < 0.0001)]. Moreover, Tukey's post‐hoc analysis showed a significant decrease in miR‐33‐5p expression level in the BDL + Nalx group compared to the BDL group (*P* < 0.0001) (Figure 8).

### 3.8 BDL Induction and the Nalx Injection Caused Histopathological Changes in the Liver and Hippocampus

Finally, we studied the effect of BDL induction and Nalx injection on the liver histopathology in rats using hematoxylin/eosin and Masson's trichrome staining. In the sham group, the liver showed normal microarchitecture and was made up of the hepatocytes aligned in rows extended radially from the centrilobular vein towards the lateral. The sinusoids were in between the cellular rows. The portal space in the periphery of the lobule consisted of the biliary arteries, veins and ducts.

Conversely, liver sections from the BDL group exhibited abnormal architecture, consisting of vague cords of hepatocytes and severe hyperplasia of biliary ducts. The dilatation or congestion of sinusoids was another finding in this group. Moreover, the inflammatory cells were seen inside the portal space and sometimes outside of it and in between the hepatocytes. The coagulative necrosis was seen as irregular necrotic patches with some inflammatory cells penetrating in the periphery. In Masson's trichrome staining, some degree of liver fibrosis was also seen.

Interestingly, in the BDL+ Nalx group, the tissue architecture was conserved to some extent. Nalx attenuated BDL‐induced liver lesions to the extent that all changes that occurred in the BDL group were seen with lesser degrees compared to the BDL group. Contrary to the BDL group, hyperplasia of the biliary ducts was seen with a mild degree. Sinusoidal distension was less severe. Milder degrees of fibrosis, inflammation, and necrosis were seen. Few coagulative patches were seen (Figure 9A).

FIGURE 9
**(A)** The effect of BDL and BDL + Nalx on the histopathology of liver. In the sham group, the liver showed normal microarchitecture and was made up of the hepatocytes aligned in rows extended radially from the centrilobular vein towards the lateral. The sinusoids were in between the cellular rows. The portal space in the periphery of the lobule consisted of the biliary arteries, veins and ducts. Conversely, liver sections from the BDL group exhibited abnormal architecture, consisting of vague cords of hepatocytes and severe hyperplasia of biliary ducts (black arrowhead). The dilatation or congestion of sinusoids was another finding in this group. Moreover, the inflammatory cells were seen inside the portal space and sometimes outside of it and in between the hepatocytes (white arrowhead). The coagulative necrosis was seen as irregular necrotic patches with some inflammatory cells penetrating in the periphery. Some degree of liver fibrosis was also seen (yellow arrows). In the BDL + Nalx group, the tissue architecture was conserved to some extent. Nalx attenuated BDL‐induced liver lesions to the extent that all changes occurred in the BDL group were seen with lesser degrees compared to the BDL group. Contrary to the BDL group, hyperplasia of the biliary ducts was seen with a mild degree. Sinusoidal distension was less severe. Milder degrees of fibrosis, inflammation and necrosis were seen. Few coagulative patches were seen. Hematoxylin/eosin and Masson's trichrome staining. Magnification 10x. Scale bar: 0.02 mm and **(B)** The effect of BDL and BDL + Nalx on the histopathology of hippocampus (Hippo.) with different magnifications in general view (× 4, and × 10) and distinct features of dentate gyrus (DG) along with its granular layer (black arrowhead), CA3, and CA1, regions in magnified view (× 40) for all groups. Sham group (first upper row): the usual microarchitecture. Pyramidal cells were uniform in size, consisting of rounded central vesicular prominent nuclei. The pyramidal layer of the CA3 (yellow arrowhead) was thicker than that of the CA1 (white arrowhead) one. The molecular layers of all the regions mostly consisted of glial cells with neuronal processes in between. The DG region also included three molecular, granular and polymorph layers. The granular layer cells were seen with small dark nuclei arranged in layers. BDL group (middle row): some changes were observed in CA3 and DG. While in the CA3 region, the cells showed no uniformity, hypochromic nuclei were seen in most cells and even in the pyknotic cells. Some regions devoid of cells were seen in this layer. The cells with central vesicular nuclei were disperse. Cellular vacuolation was seen in the granular layer of the DG. Some regions devoid of cells were observed in this layer, as well. The presence of hypochromic and pyknotic nuclei in cells could be indicative of cell death in this layer. BDL + Nalx (lower row): the pyramidal layer of the CA3 was more similar to the control group. The count of the cells with pyknotic and hypochromic nuclei was lower than in the BDL group. Nonetheless, a large number of rounded vesicular cells were seen more in this group. The granular layer of the DG was more similar to the BDL group. Although less hypochromic cells were seen, cellular vacuolation and regions devoid of cells were also seen. **Abbreviations**: BDL, bile duct ligation; CA1, Cornu Ammonis 1; CA3, Cornu Ammonis 3; DG, dentate gyrus; Hippo., hippocampus; Nalx, naloxone. Scale bar: 0.5 and 0.02 mm and for Hippo. (4x and 10x, respectively), and 0.05 mm for remaining columns.

**Figure:**
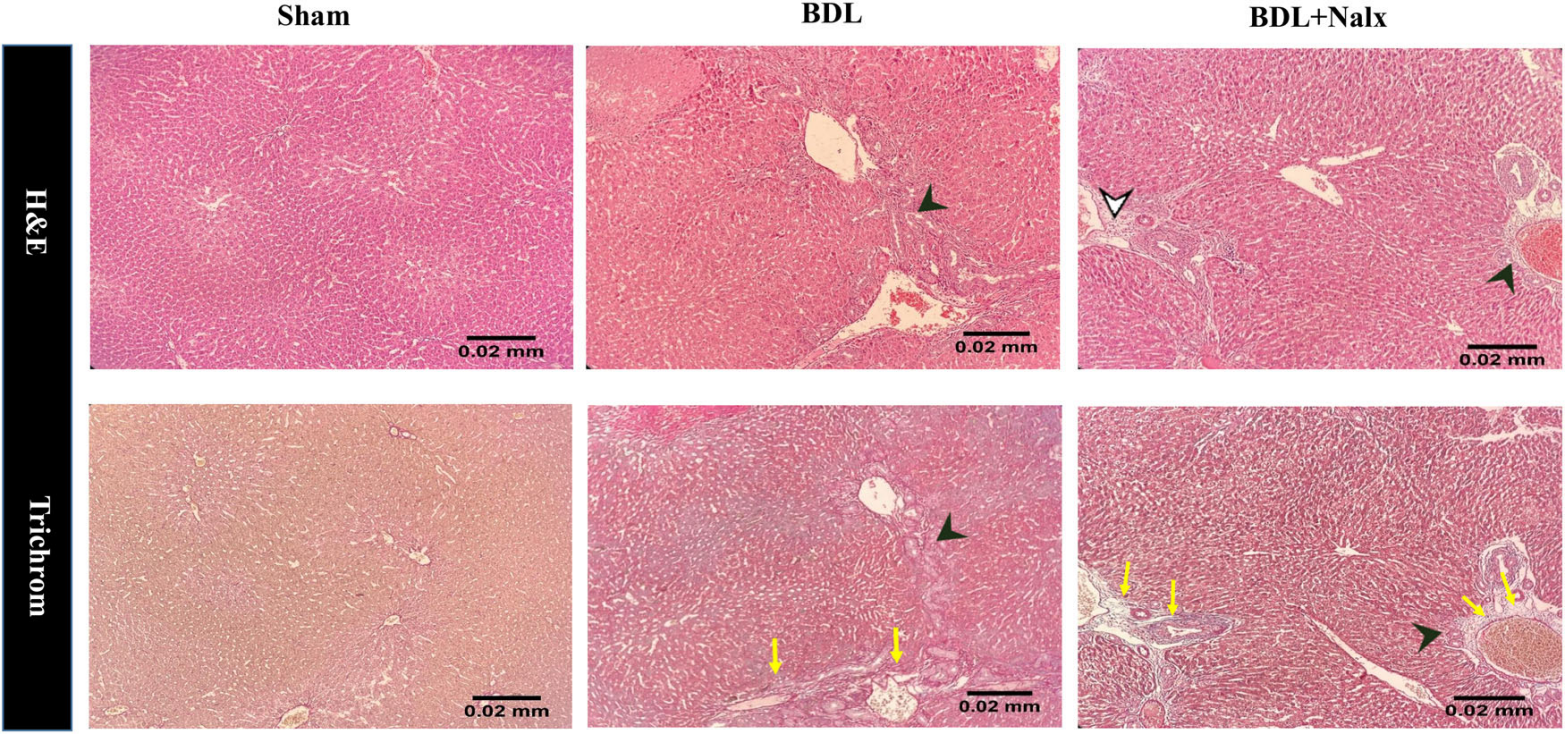

**Figure:**
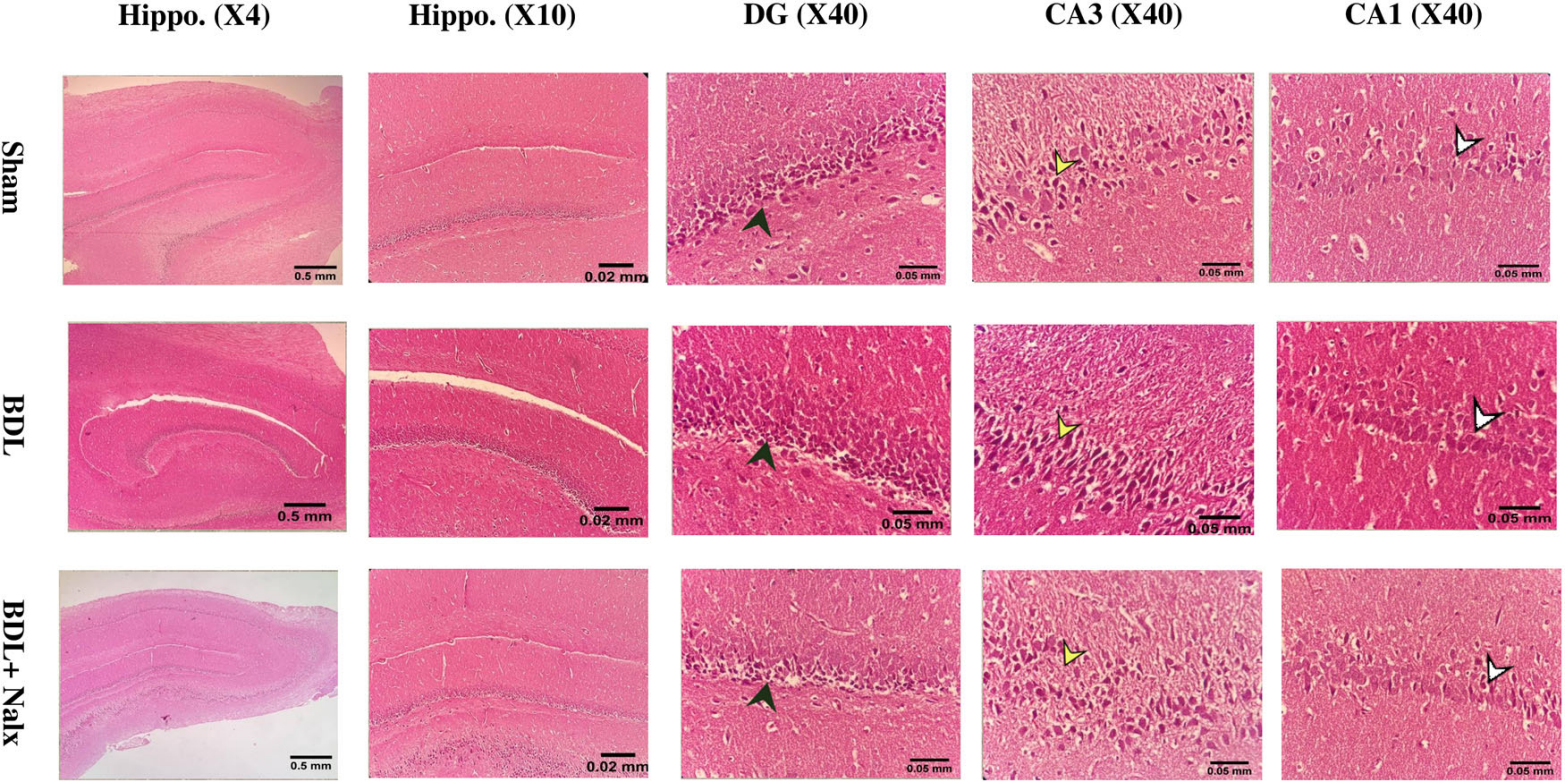

The hippocampi of the sham group showed the usual microarchitecture and were made up of the CA1 and CA3 regions, including molecular, pyramidal, and polymorphic layers. Pyramidal cells were uniform in size, consisting of rounded, central, vesicular, prominent nuclei. The pyramidal layer of the CA3 was thicker than that of the CA1. The molecular layers of all the regions mostly consisted of glial cells with neuronal processes in between. The dentate gyrus (DG) region also included three molecular, granular, and polymorph layers. The granular layer cells were seen with small dark nuclei arranged in layers.

In the BDL group, some changes were observed in CA3 and DG. While in the CA3 region, the cells showed no uniformity; hypochromic nuclei were seen in most cells and even in the pyknotic cells. Some regions devoid of cells were seen in this layer. The cells with central vesicular nuclei (similar to the cells in the control group) were dispersed. Cellular vacuolation was seen in the granular layer of the DG. Some regions devoid of cells were observed in this layer as well. The presence of hypochromic and pyknotic nuclei in cells could be indicative of cell death in this layer.

In the BDL+ Nalx group, the pyramidal layer of the CA3 was more similar to the control group. The count of the cells with pyknotic and hypochromic nuclei was lower than in the BDL group. Nonetheless, a large number of rounded vesicular cells (similar to the major cells of the pyramidal layer in the control group) were seen more in the BDL+ Nalx group. The granular layer of the DG was more similar to the BDL group. Although fewer hypochromic cells were seen, cellular vacuolation and regions devoid of cells were also seen (Figure 9B).

## 4 Discussion

Previous studies have shown that miRs contribute to liver diseases and play a crucial role in various neurological diseases, such as memory disorders (see the introduction). To identify specific therapeutic strategies for the treatment of such neurological disorders, various molecular signaling approaches, such as functional studies of non‐coding RNA, have been conducted. Meanwhile, the precise contribution of miR‐33‐5p to cognitive dysfunction in HE remains largely unknown. This study explored the miR‐33‐5p expression profiles as well as neurocognitive and histopathological changes in rats with normal and bile duct‐ligated liver and hippocampus tissues to address the subject. Further research on these non‐coding RNAs may lead to new therapies for the treatment and management of brain dysfunction caused by bile duct obstruction. Likewise, the same mechanisms would contribute to some of the cognitive alterations in patients with biliary obstruction pathologies. Hence, biochemical BDL‐induced metabolic changes in the brain may be involved in cognitive decline.

Furthermore, previous biochemical mechanistic studies of the association between HE and memory dysfunction have mainly investigated the role of liver function tests. In line with the studies showing the elevation of liver function test results as criteria for the establishment of an HE model (Ganjalikhan‐hakemi et al. 2023; López‐Velázquez et al. 2014; Mohammadian et al. 2019; Škvareninová and Kostecká 2023; Tamnanloo et al. 2023), except for GGT, we observed a similar increasing pattern in the levels of ALP, ALT, AST, Bil.D, and Bil. T and LDH. The GGT failure to increase, despite the induction of the model, may be attributed to the strict time window for the measurement of GGT. Previous studies have reported that GGT increase occurs in the range of 28 (Teixeira et al. 2013) to 35 days (Eshraghi et al. 2015). Nonetheless, the finding that serum GGT did not elevate acutely (>24 h) but elevated chronically (during 2, 4, and 6 weeks), may be due to the different methods used for the induction of the BDL model.

In agreement with the fact that neurobehavioral consequences of BDL are well documented in studies (Felipo 2013; Zhou et al. 2016), the impairment of IA memory following BDL was also confirmed in the present study. We observed that the IA memory of animals was impaired 14 days following BDL. Although the exact mechanisms responsible for the impairment in learning and memory abilities are beginning to be clarified, the available evidence shows that endogenous opioid levels are elevated in human and animal BDL models (see the introduction). Accordingly, rats with BDL revealed memory impairment starting on day 14, persisting for at least the first 28 days (Javadi‐Paydar et al. 2013). Nevertheless, Nasehi et al. (2013) reported that memory acquisition in cholestatic mice did not change seven days following BDL (Nasehi et al. 2013). Meanwhile, it was demonstrated in Javadi‐Paydar's study that cholestasis causes a progressive increase in endogenous opioid levels, which affects memory function (Javadi‐Paydar et al. 2013). In our study, Nalx to some extent reversed some cognitive and pathological changes induced by BDL. Considering that changes occurred following bile duct obstruction to manifest the HE, it needs a longer time; however, the time period of our model may not be able to completely mimic the HE model and so correct all changes seen in HE, and the Nalx was effective in the cholestasis stage of the model. Moreover, a large body of evidence shows that the μ‐opioid receptor antagonist, Nalx, can alleviate memory impairment caused by opioids (e.g., morphine) during several learning and memory phases (Castellano et al. 1994; Homayoun et al. 2003), indicating the involvement of μ‐opioid receptors in the mechanism. Long‐term changes occurred during the HE process, which needs repetitive applications of Nalx to antagonize and reverse all changes, though in our memory studies, using a single dose of Nalx in the BDL+ Nalx group could alleviate IA memory impairment and reverse some changes. However, in the Zarrindast et al. (2020) study, three doses of Nalx were used, and in the Afshari et al. (2018) study, both the acute dose and the chronic one‐week microinjection of Nalx were applied (Afshari et al. 2018; Zarrindast et al. 2020).

In addition, following the neurobehavioral investigation, we observed no changes in locomotion; however, as Zarrindast et al. (2020) reported, the increased locomotor activities in BDL mice with the same dose of Nalx (2 mg/kg) potentially confirm an increase in endogenous opioids and opioid receptor activation throughout this procedure (Zarrindast et al. 2020).

In addition to the neurobehavioral consequences of BDL, some findings reported electrophysiological correlates of HE for its cognitive significance. We recorded LTP as a well‐known hallmark of synaptic plasticity and cellular mechanism of learning and memory in the hippocampus. Long‐term synaptic plasticity is a leading candidate for the cellular mechanism underlying learning and memory (Mohammadian et al. 2019). The induction of LTP is modulated through AMPA and NMDA receptors in the hippocampus and the subsequent activation of the glutamate‐nitric oxide‐cGMP pathway (França et al. 2019). In agreement with previous research showing impaired LTP in BDL animals (Mohammadian et al. 2019), we observed the disruption of LTP in the BDL group. Moreover, in Hajipour's study, it was demonstrated that the induction of an experimental acute HE model caused spatial memory impairment and hippocampal LTP deficit and the disruption of some major serum biochemical and liver enzymes and oxidative stress in the brain (Hajipour et al. 2021). As reported in previous studies, the development of normal long‐term memory and neuronal plasticity are two physiological processes that are extensively impaired by such severe disruption (i.e., BDL) in neuronal function (Mohammadian et al. 2019; Shabani et al. 2019). Additionally, we also noticed the rightward and downward shift of the input/output curve in the BDL group, which is in line with the findings of experiments indicating a reduction in the basal excitability of hippocampal CA1 neurons (Aghaei et al. 2016; Mohammadian et al. 2019; Tahamtan et al. 2017). Furthermore, as an inherent electrophysiological characteristic of the hippocampus neurons, some researchers have previously demonstrated that BDL dramatically reduces baseline neuronal excitability, which may be attributed to increases in *I*_A_, *K*_Ca2+_, and Ca^2+^ currents (Shabani et al. 2019; Tahamtan et al. 2017). Accordingly, in terms of the clinical application of the changes seen in the animal models of HE, the study by Golaszewski (2016) offered the first in vivo electrophysiological proof that minimal hepatic encephalopathy patients also have altered LTP (Golaszewski et al. 2016). The aforementioned “subclinical” signs of such patients may manifest the functional correlates of the anomalies seen in LTP‐like mechanisms. Such patients have reduced associative sensorimotor plasticity as an indirect probe of motor learning (Golaszewski et al. 2016).

Likewise, alterations in miRs and their downstream signaling pathways may also play a role in the induction and progression of cognitive dysfunction observed during HE. The findings taken from a previous study confirmed the miR‐33‐dependent suppression of bile salt exporter in hepatocytes (Rayner et al. 2010). It has been noted that bile salt exporters are the direct targets of the *miR‐33* and its overexpression inhibits bile salt transporters, lowering plasma high‐density lipoprotein levels and hence regulating bile formation and secretion (Allen et al. 2012; Sidorkiewicz 2023).

Nevertheless, it has also been documented that a range of morphine‐regulated miRs influence the learning, memory, and plasticity processes by modifying the μ‐opioid receptor (Hwang et al. 2012; Vastegani et al. 2022; Wu et al. 2009; Zheng and Chu et al. 2010). In addition, antagonizing miRs associated with the μ‐opioid receptor also signifies the involvement of the opioid mechanism (Hwang et al. 2012). As a result, the evidence for miRs' modulatory function in opioid physiology is continuously growing. Due to the fact that endogenous opioids increase in BDL, in the current study, we observed the overexpression of miR‐33‐5p following BDL induction. The finding is in line with the results of our previous work showing that morphine up‐regulates the hippocampal miR‐33‐5p expression (Vastegani et al. 2022). Considering the enormous body of evidence emphasizing the functional connections between miRs and the opioidergic system (Guo et al. 2002; He et al. 2010; Wu et al. 2009; Zheng and Chu et al. 2010; Zheng et al. 2010) and the notion that endogenous opioid levels are elevated in human and animal BDL models (see the introduction), it is reasonable to assume the involvement of miR‐33‐5p in the cholestasis process, as was the case for the results seen in the BDL + Nalx group. In the mentioned procedure, the administration of Nalx antagonized the miR‐33‐5p overexpression induced by BDL. This is in agreement with our previous report showing that miR‐33‐5p controls the morphine‐dependent gene expression, and the modifications in miR‐33‐5p expression may therefore have a negative impact on memory and learning (Vastegani et al. 2022).

Ultimately, in the final series of our experiments, we used a qualitative histopathological analysis to find the pathophysiological mechanisms of BDL on cognition. The histological changes of liver tissue in HE are well documented (Cho et al. 2020; Eshraghi et al. 2015; Hajipour et al. 2021; Teixeira et al. 2013). As previously reported (Liu et al. 2018), HE leads to noticeable changes in the liver tissue architecture with the appearance of necrotic hepatocytes and inflammatory processes, mainly macrophages, around the central vein. Remarkably, in the Nalx‐treated group, Nalx attenuated BDL‐induced liver lesions to the extent that all changes occurring in the BDL group were seen with minor grades. Consistent with the neurobehavioral fact that Nalx can reverse BDL‐induced memory disorders (Nasehi et al. 2013), the available indirect evidence supports that Nalx could ameliorate morphine‐induced histopathological insults (Peirouvi et al. 2020); the application of opioid antagonists prevented the development of hepatic fibrosis in cirrhosis (Ebrahimkhani et al. 2006), and the BDL‐induced histological changes were reversed after the injection of an opioid antagonist (Rahimi et al. 2018).

Consistent with the findings from the hippocampal sections, the BDL rat model of HE showed a notably large number of injured neurons in the CA1 area of the hippocampus (Ganjalikhan‐hakemi et al. 2023). In addition, microglia activation and subsequent neuroinflammation are seen within the hippocampal subfields CA1, CA3, and DG (El‐Mansoury et al. 2024) following HE. Moreover, a thioacetamide‐induced HE also resulted in neuroinflammation with regard to microglial activation, causing neurodegeneration in the CA1 subfield (Adelodun et al. 2024). A study using immunohistochemistry in a chronic rat HE model showed that hippocampal CA1 pyramidal neurons have reduced spine density (Chen et al. 2014). The swelling of astrocytes and the multiplication of microglia may raise ambient pressure, which may lead to a reduction in cortical pyramidal neurons' dendritic spines. Indeed, adult synaptic remodeling is influenced by the interaction between microglia and synapses (Ji et al., 2013). Since there is evidence that hyperammonemia may increase reactive oxygen and nitrogen oxide species in astrocytes, oxidative stress may also be a component contributing to the decline in the dendritic spines of cortical pyramidal neurons(Lachmann et al. 2013). Research indicates that liver disorders and HE are associated with decreased neurogenesis and neuroplasticity, especially when hyperammonemia is present (Chen et al. 2014; Yang et al. 2015).

## 5 Conclusions

The present study shows that BDL‐induced memory impairment has a molecular, electrophysiological and histopathological basis and might be regulated by miR‐33‐opioid signaling. Nonetheless, further research is needed to disclose the precise mechanistic significance of the above signaling and its involvement in the cognitive pathophysiology associated with BDL. Thus, we suggest that the modulation of miR in the brain may be a novel therapeutic approach for relieving neurohepatic pathologies and emphasize the potential ameliorative role of Nalx. Therefore, individuals with cholestasis may benefit from new therapeutic approaches that focus on this brain circuit in terms of cognitive and synaptic plasticity.

## Author Contributions

**Mohadeseh Rahimi‐vala**: acquisition. **Behrang Alani**: acquisition, analysis, supervision, and interpretation of data. **Ali Arjmand**: writing – original draft preparation. **Tahere Mazoochi**: acquisition. **Abolfazl Ardjmand**: conceptualization, methodology and design of the work, reviewing and editing, supervision, analysis and interpretation of data, supervision, project administration, funding acquisition and resources.

## Ethics Statement

The 2011 Guide for the Care and Use of Laboratory Animals developed by the National Academy of Sciences' Institute for Laboratory Animal Research served as the basis for all the procedures. The study protocol (IR.KAUMS.AEC.1401.008) was approved by Kashan University of Medical Sciences (KAUMS) Research Ethics Committee (REC).

## Data Availability

The datasets generated during and/or analyzed during the current study are available from the corresponding author upon reasonable request. The primer sequences for the microRNA qPCR are proprietary to Anacellteb, Tehran, Iran (Ana microRNA) and are not publicly available.
